# Supplementary material for: Vertically optimized phase separation with improved exciton diffusion enables efficient organic solar cells with thick active layers
Source: Nat Commun. 2022 May 2;13:2369. doi: 10.1038/s41467-022-29803-6 (PMC9061803; doi:10.1038/s41467-022-29803-6)
Supplement: Supplementary file 1 — Supplementary Information [file 41467_2022_29803_MOESM1_ESM.pdf]

SUPPLEMENTARY INFORMATION

Vertically Optimized Phase Separation with Improved Exciton Diffusion Enables Efficient Organic Solar Cells with Thick Active Layers

Cai et al.

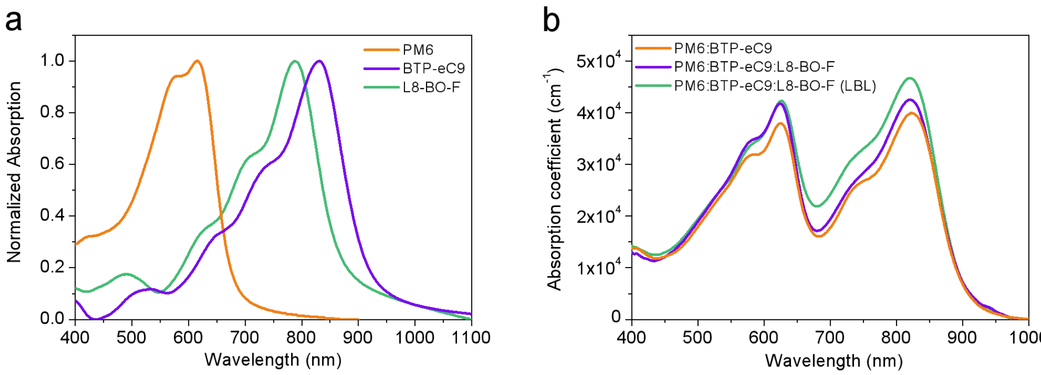

Supplementary Figure 1. UV-vis absorption spectra of **a** pristine PM6, BTP-eC9, and L8-BO-F films, and **b** 120 nm-thick PM6:BTP-eC9 binary and PM6:BTP-eC9:L8-BO-F ternary films.

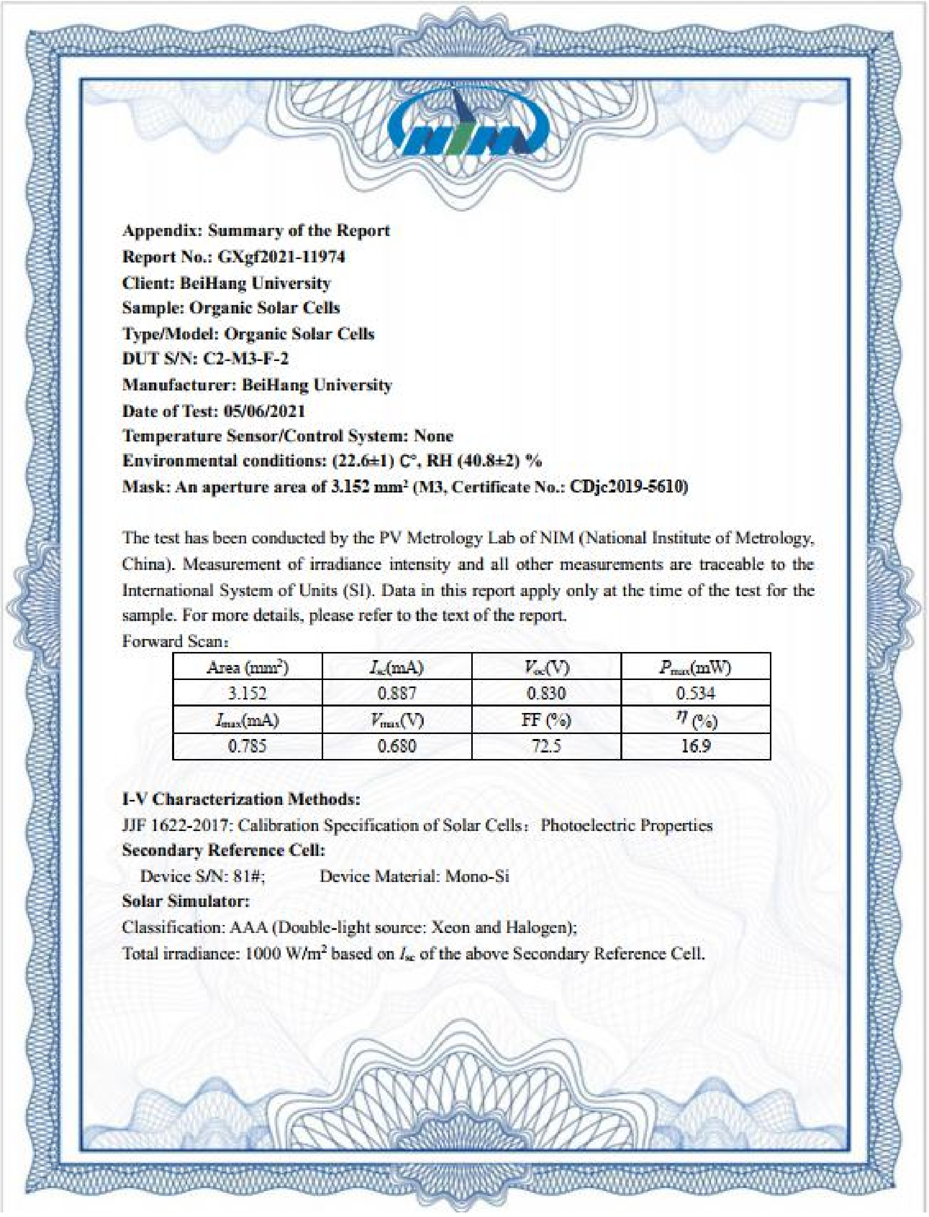

Appendix: Summary of the Report
Report No.: GXgf2021-11974
Client: BeiHang University
Sample: Organic Solar Cells
Type/Model: Organic Solar Cells
DUT S/N: C2-M3-F-2
Manufacturer: BeiHang University
Date of Test: 05/06/2021
Temperature Sensor/Control System: None
Environmental conditions: (22.6±1) C°, RH (40.8±2) %
Mask: An aperture area of 3.152 mm² (M3, Certificate No.: CDjc2019-5610)

The test has been conducted by the PV Metrology Lab of NIM (National Institute of Metrology, China). Measurement of irradiance intensity and all other measurements are traceable to the International System of Units (SI). Data in this report apply only at the time of the test for the sample. For more details, please refer to the text of the report.

Forward Scan：

Area (mm^2)	I_{sc}(mA)	V_{oc}(V)	P_{max}(mW)
3.152	0.887	0.830	0.534
I_{max}(mA)	V_{max}(V)	FF (%)	η (%)
0.785	0.680	72.5	16.9

I-V Characterization Methods:
JJF 1622-2017: Calibration Specification of Solar Cells：Photoelectric Properties
Secondary Reference Cell:
Device S/N: 81#; Device Material: Mono-Si
Solar Simulator:
Classification: AAA (Double-light source: Xeon and Halogen);
Total irradiance: 1000 W/m² based on I_{sc} of the above Secondary Reference Cell.

Supplementary Figure 2. The certification report for LBL-processed PM6:BTP-eC9:L8-BO-F device with 300 nm active layer thickness from National Institute of Metrology (NIM), China.

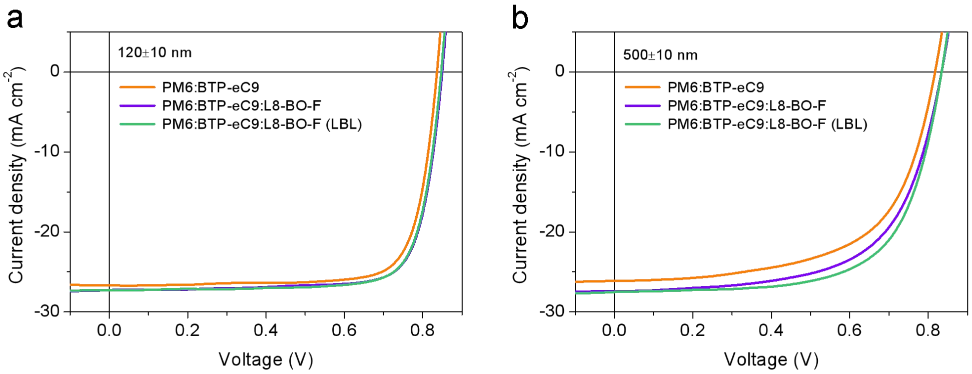

Supplementary Figure 3. *J-V* characteristics of PM6:BTP-eC9 and PM6:BTP-eC9:L8-BO-F devices with active layer thicknesses of **a** 120 nm, **b** 500 nm under simulated AM 1.5 G illumination at 100 mW cm^{-2}

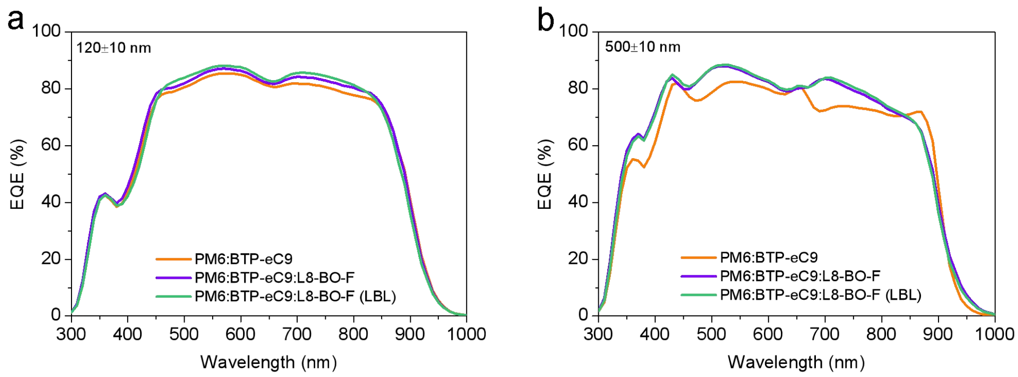

Supplementary Figure 4. EQE curves of PM6:BTP-eC9 and PM6:BTP-eC9:L8-BO-F devices with active layer thicknesses of **a** 120 nm, and **b** 500 nm.

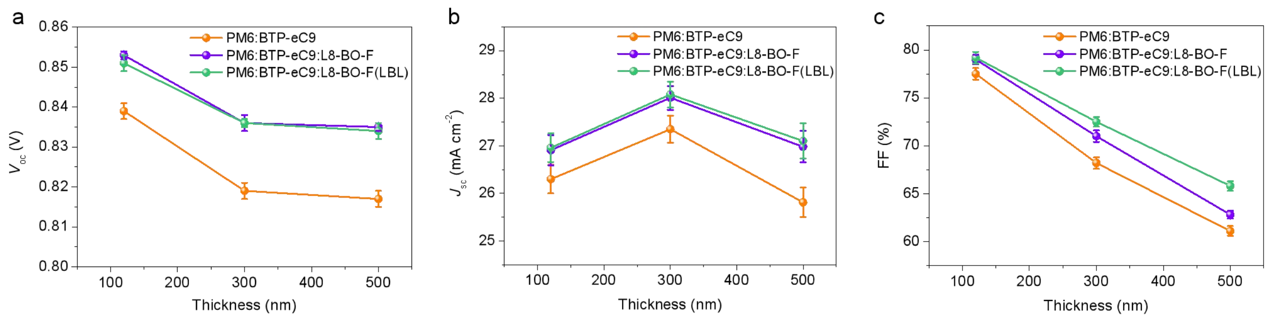

Supplementary Figure 5. Dependence of **a** V_{oc}, **b** J_{sc}, and **c** FF on the active layer thickness. In all figures, the error bars represent the standard deviations.

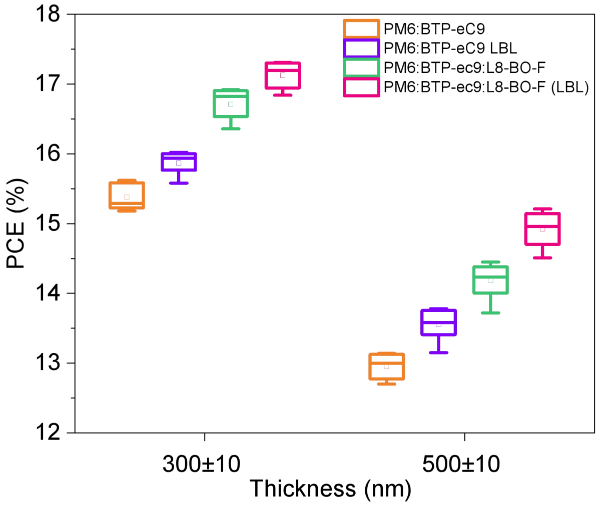

Supplementary Figure 6. Comparison of PCEs of the conventional and LBL-processed PM6:BTP-eC9 and PM6:BTP-eC9:L8-BO-F devices with different thicknesses. The box, horizontal line, and square symbol denote 25/75 percentile, the median value, and mean value respectively. The sample size for each column is 20 devices.

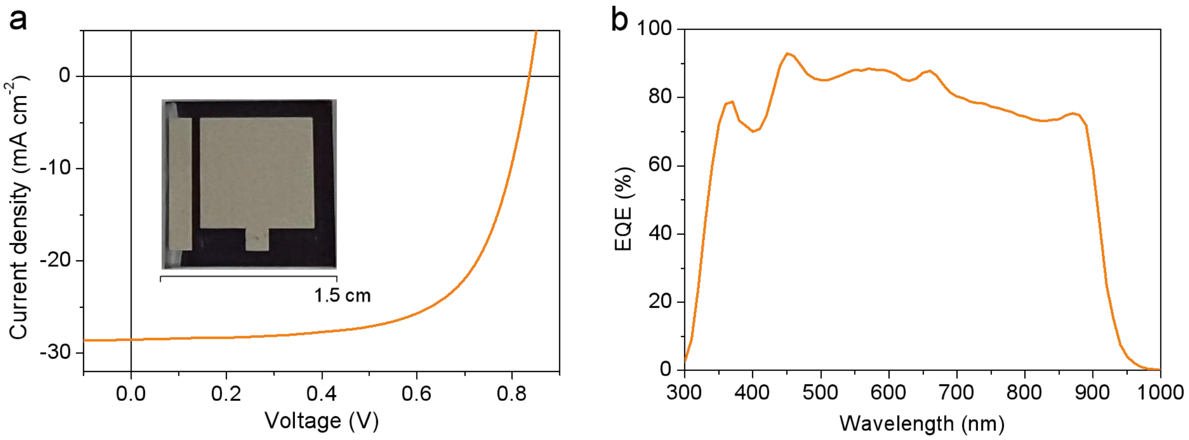

Supplementary Figure 7. **a** *J-V* curve of LBL-processed ternary OSC with 1 cm^2 active area, and **b** the corresponding EQE spectrum. The inset is a photograph of the real large-area device.

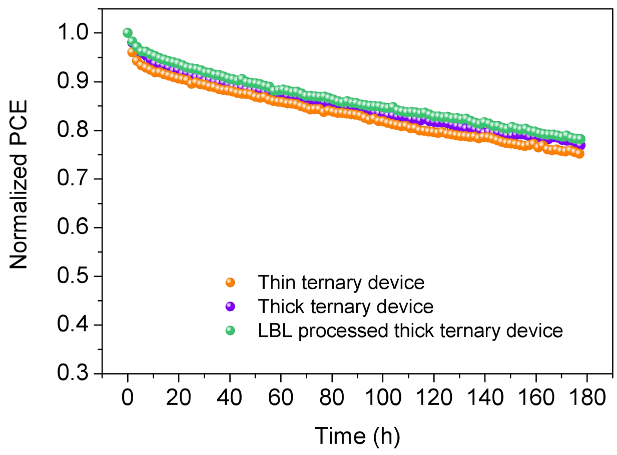

Supplementary Figure 8. Photostability of 120 nm- and 300 nm-thick ternary devices measured under continuous 1 sun illumination in air.

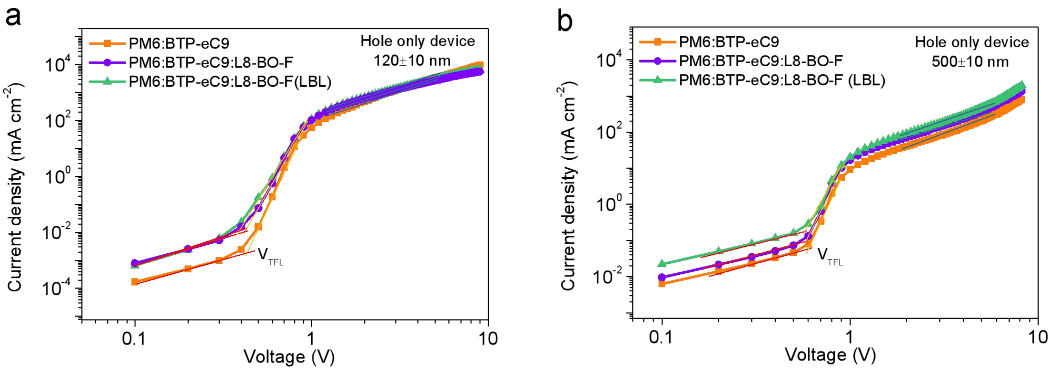

Supplementary Figure 9. *J-V* characteristics of hole-only devices based on PM6:BTP-eC9 and PM6:BTP-eC9:L8-BO-F blends with **a** 120 nm and **b** 500 nm thicknesses.

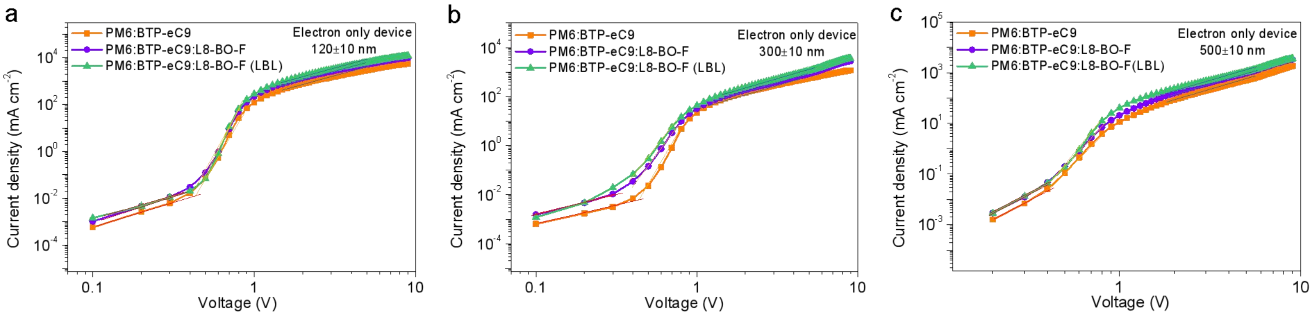

Supplementary Figure 10. *J-V* characteristics of electron-only devices based on PM6:BTP-eC9 and PM6:BTP-eC9:L8-BO-F blends with **a** 120 nm, **b** 300 nm, and **c** 500 nm thicknesses.

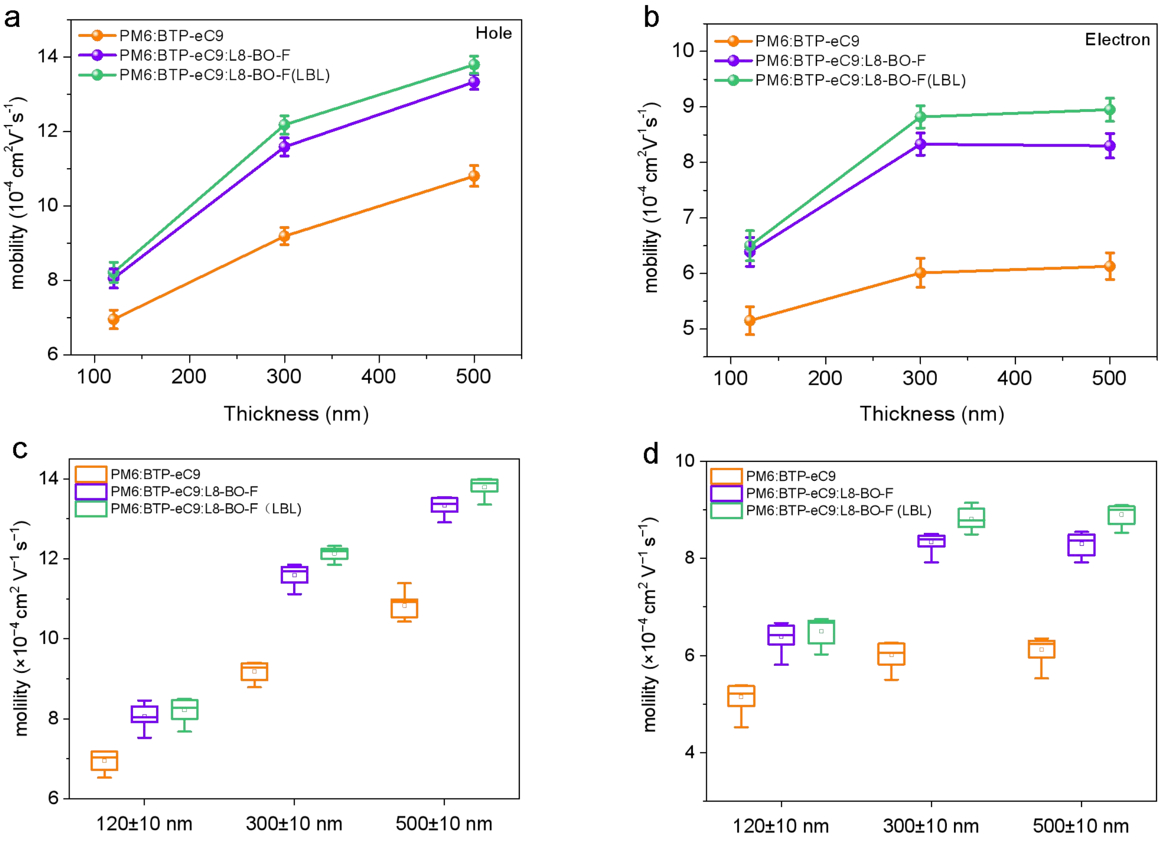

Supplementary Figure 11. a Dependence of hole mobilities on the active layer thickness, and **b** dependence of electron mobilities on the active layer thickness. The error bars represent the standard deviations. **c, d** Comparison of the hole (**c**) and electron (**d**) mobilities of the PM6:BTP-eC9, PM6:BTP-eC9:L8-BO-F and PM6:BTP-eC9:L8-BO-F LBL processed devices with different thicknesses. The box, horizontal line, and square symbol denote 25/75 percentile, the median value, and mean value respectively. The sample size for each column is 20 devices.

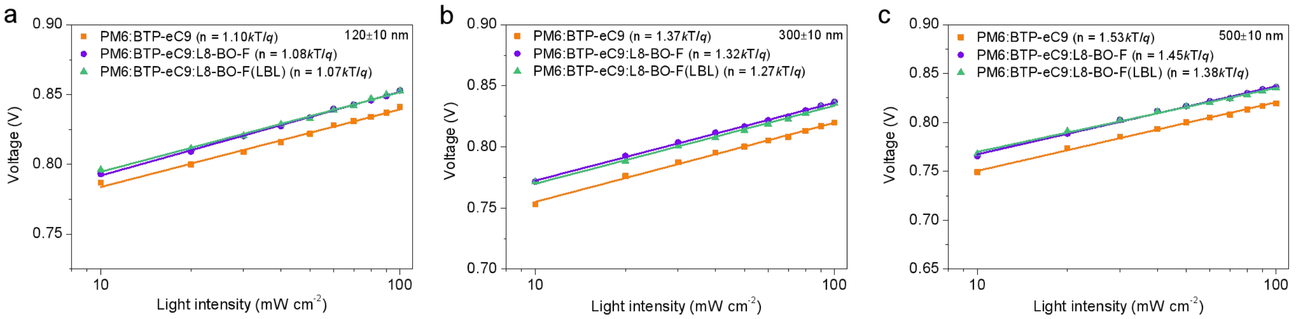

Supplementary Figure 12. V_{oc} versus light intensity of PM6:BTP-eC9 and PM6:BTP-eC9:L8-BO-F devices with active layer thicknesses of **a** 120 nm, **b** 300 nm, and **c** 500 nm.

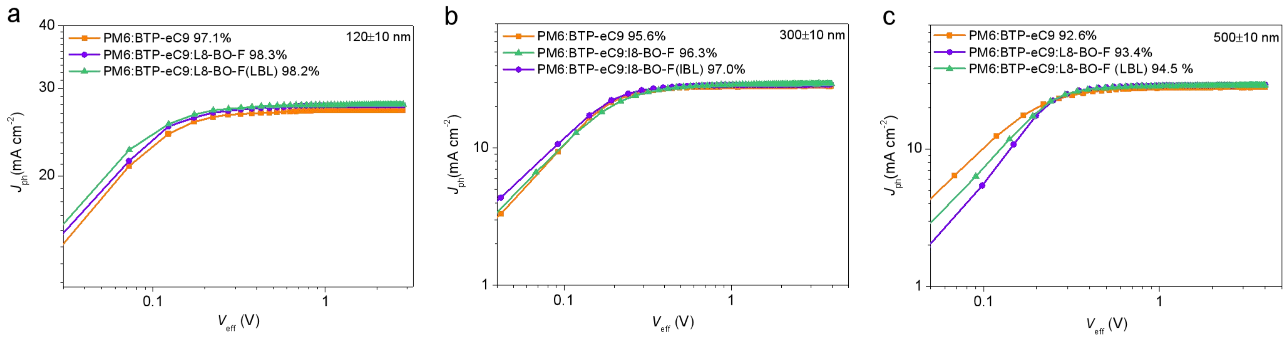

Supplementary Figure 13. Photocurrent density (J_{ph}) as a function of effective bias (V_{eff}) for PM6:BTP-eC9 and PM6:BTP-eC9:L8-BO-F devices with active layer thicknesses of **a** 120 nm, **b** 300 nm, and **c** 500 nm.

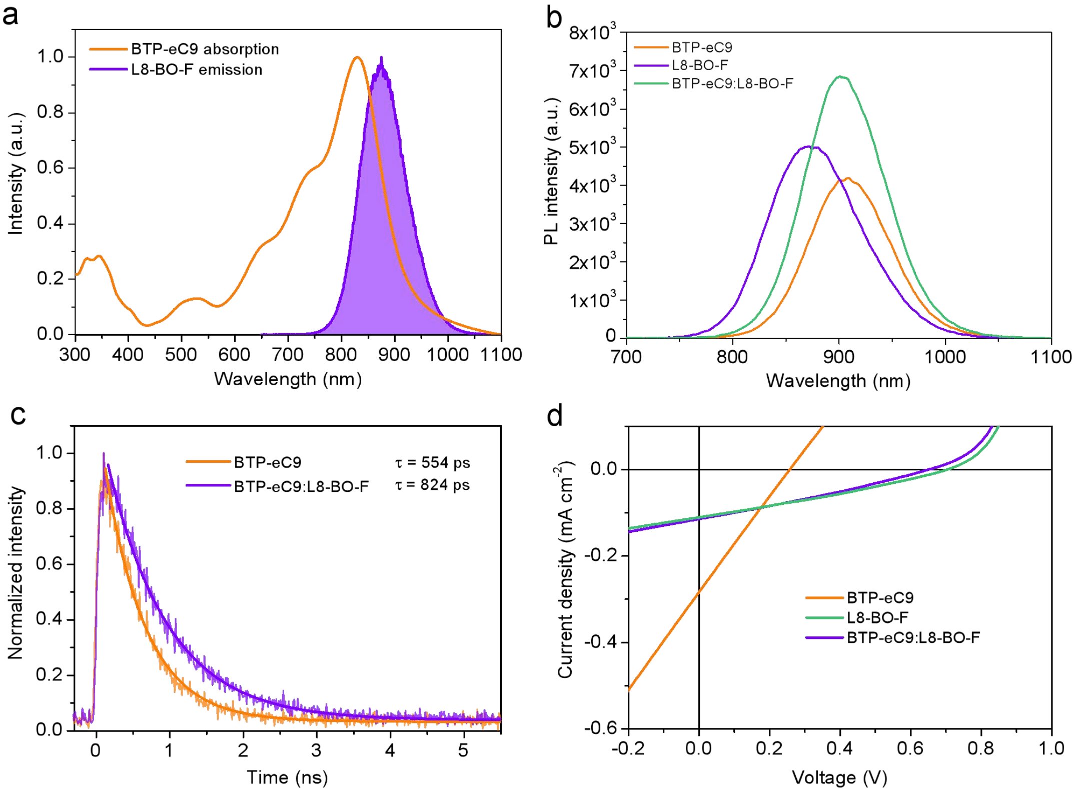

Supplementary Figure 14. a UV absorption spectrum of BTP-eC9 and PL spectrum of L8-BO-F. **b** PL spectra of BTP-eC9, L8-BO-F and BTP-eC9:L8-BO-F films. **c** TRPL spectra of BTP-eC9 and BTP-eC9:L8-BO-F films. **d** *J-V* characteristics of OSCs based on BTP-eC9, L8-BO-F and BTP-eC9:L8-BO-F films.

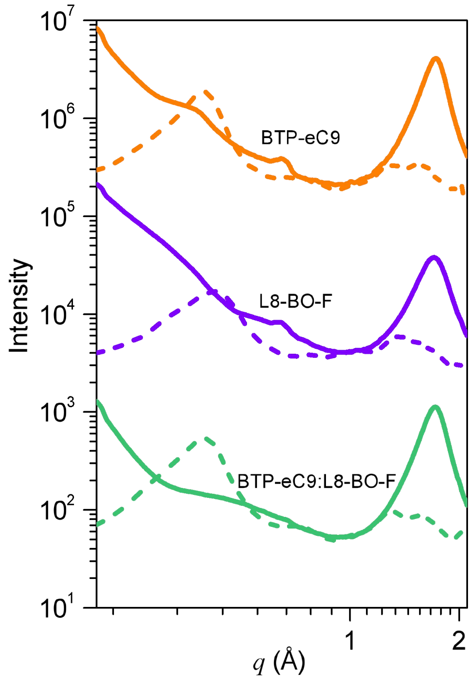

Supplementary Figure 15. In-plane (dashed lines) and out-of-plane (solid lines) line-cuts extracted from the 2D GIWAXS profiles of neat BTP-eC9, L8-BO-F and BTP-eC9:L8-BO-F films.

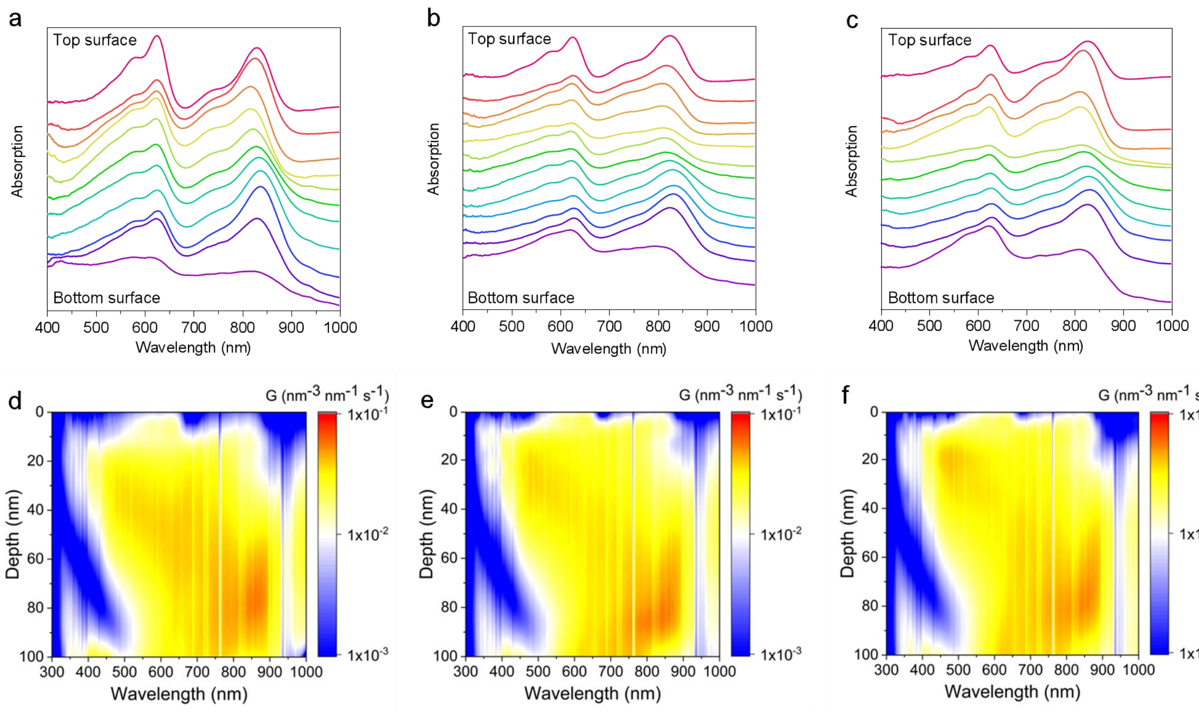

Supplementary Figure 16. a-c Film-depth-dependent light absorption spectra of **(a)** PM6:L8-BO-F blend, **(b)** PM6:BTP-eC9:L8-BO-F blend, **(c)** LBL-processed PM6:BTP-eC9:L8-BO-F blend with 120 nm thickness. **d-f** Exciton generation contours of **(d)** PM6:L8-BO-F blend, **(e)** PM6:BTP-eC9:L8-BO-F blend, **(f)** LBL-processed PM6:BTP-eC9:L8-BO-F blend, as simulated from spectra of **a-c** in combination with optical transfer-matrix approach. The "noise"-like vertical lines are due to features of the AM 1.5G solar spectra.

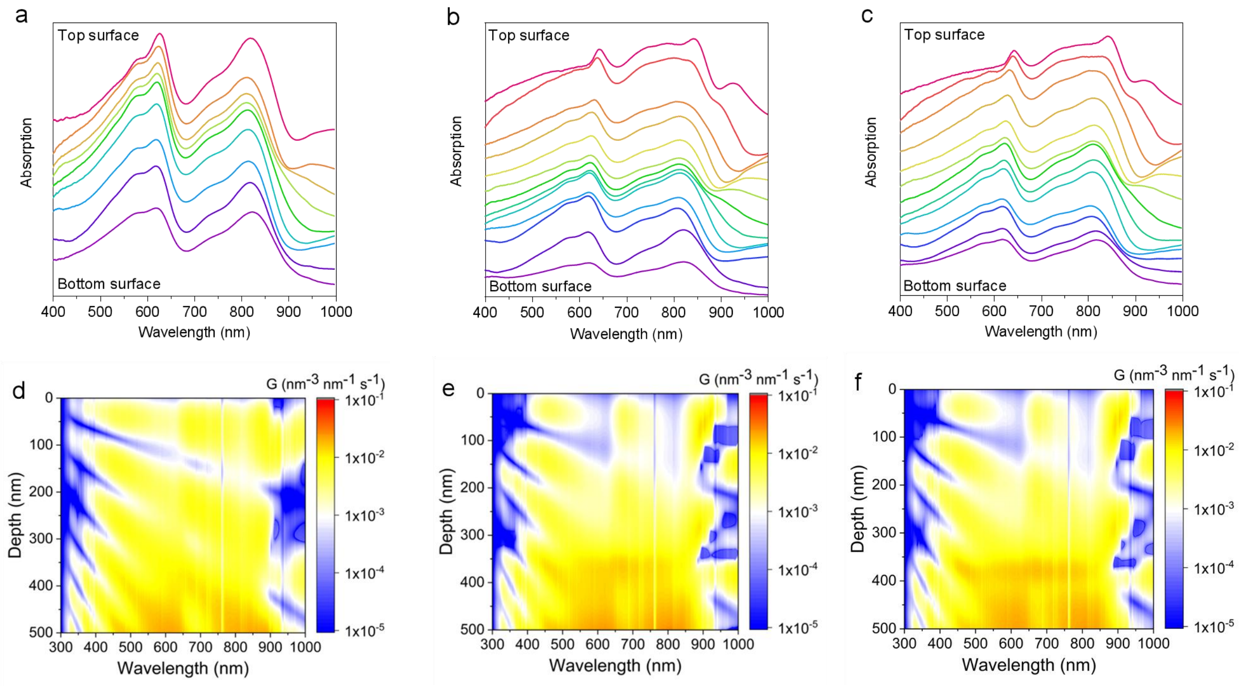

Supplementary Figure 17. a-c Film-depth-dependent light absorption spectra of **(a)** PM6:L8-BO-F blend, **(b)** PM6:BTP-eC9:L8-BO-F blend, **(c)** LBL-processed PM6:BTP-eC9:L8-BO-F blend with 500 nm thickness. **d-f** Exciton generation contours of **(d)** PM6:L8-BO-F blend, **(e)** PM6:BTP-eC9:L8-BO-F blend, **(f)** LBL-processed PM6:BTP-eC9:L8-BO-F blend, as simulated from spectra of **a-c** in combination with optical transfer-matrix approach. The “noise”-like vertical lines are due to features of the AM 1.5G solar spectra.

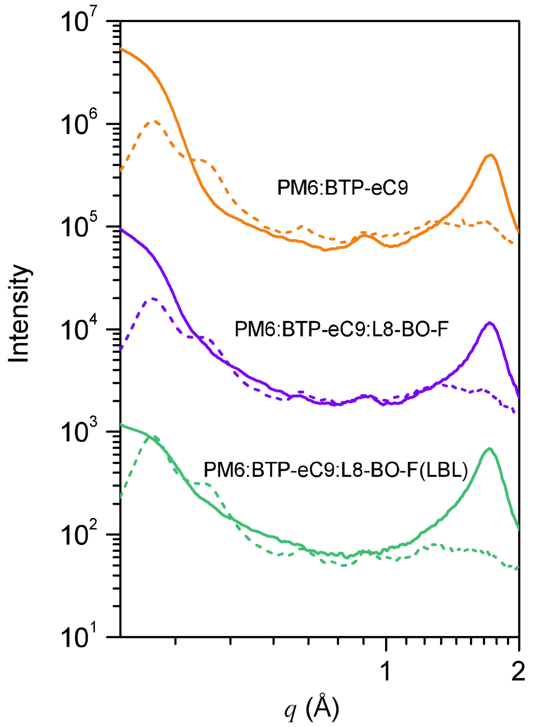

Supplementary Figure 18. In-plane (dashed lines) and out-of-plane (solid lines) line-cuts extracted from the 2D GIWAXS profiles of the PM6:BTP-eC9 and PM6:BTP-eC9:L8-BO-F blend films with 300 nm active layer thickness.

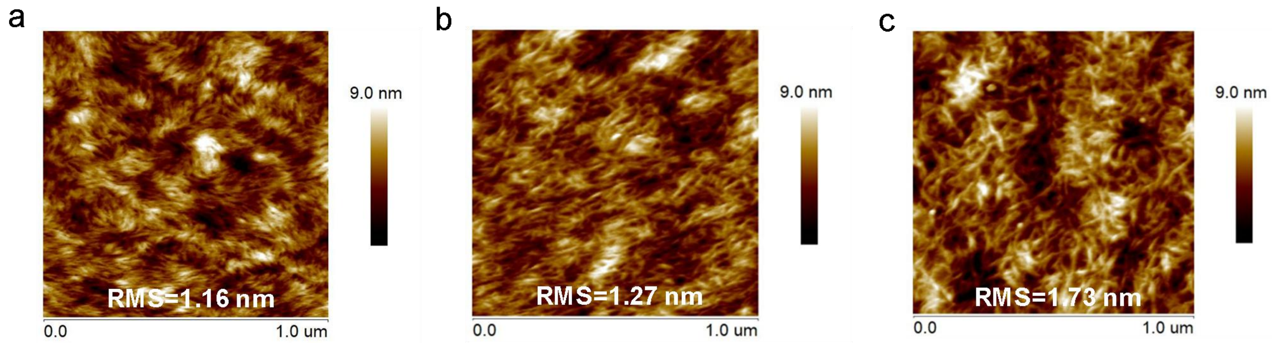

Supplementary Figure 19. AFM height images of **a** PM6:BTP-eC9, **b** PM6:BTP-eC9:L8-BO-F, and **c** LBL-processed PM6:BTP-eC9:L8-BO-F blends with 300 nm active layer thickness.

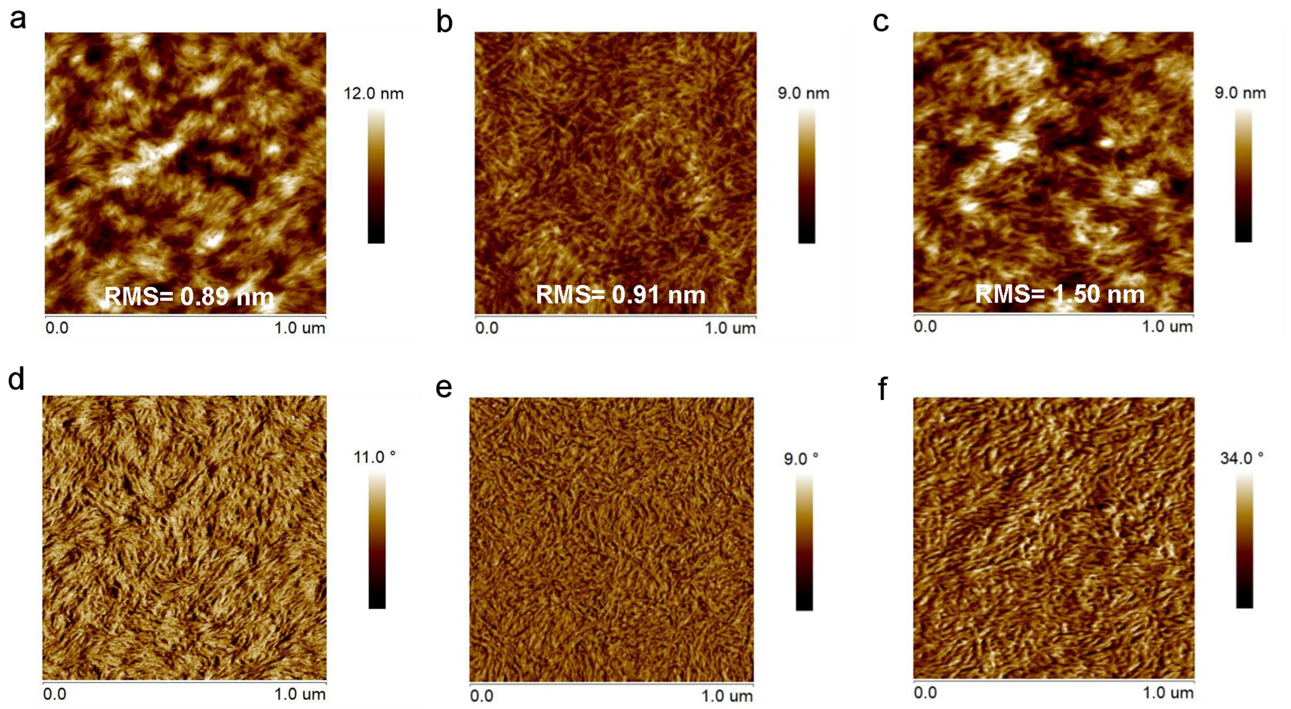

Supplementary Figure 20. AFM height images of **a** PM6:BTP-eC9, **b** PM6:BTP-eC9:L8-BO-F, **c** LBL-processed PM6:BTP-eC9:L8-BO-F blends with 120 nm active layer thickness, and **d-f** the corresponding phase images.

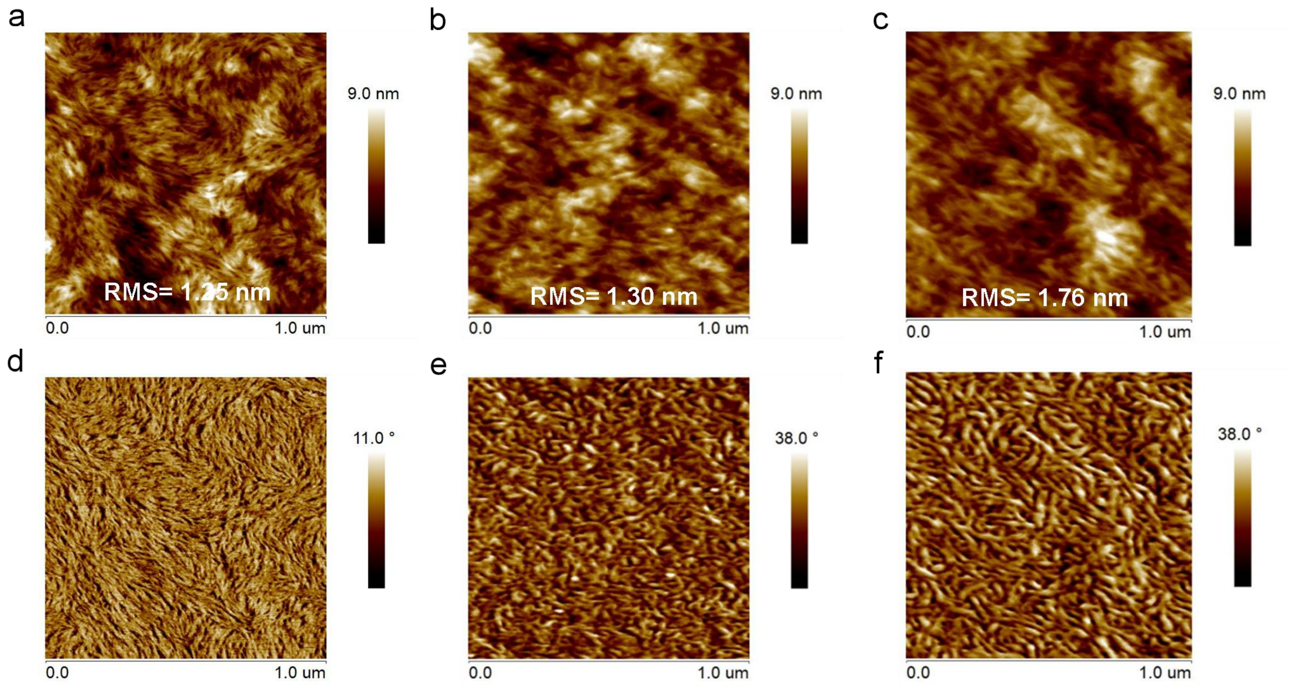

Supplementary Figure 21. AFM height images of **a** PM6:BTP-eC9, **b** PM6:BTP-eC9:L8-BO-F, **c** LBL-processed PM6:BTP-eC9:L8-BO-F blends with 500 nm active layer thickness, and **d-f** the corresponding phase images.

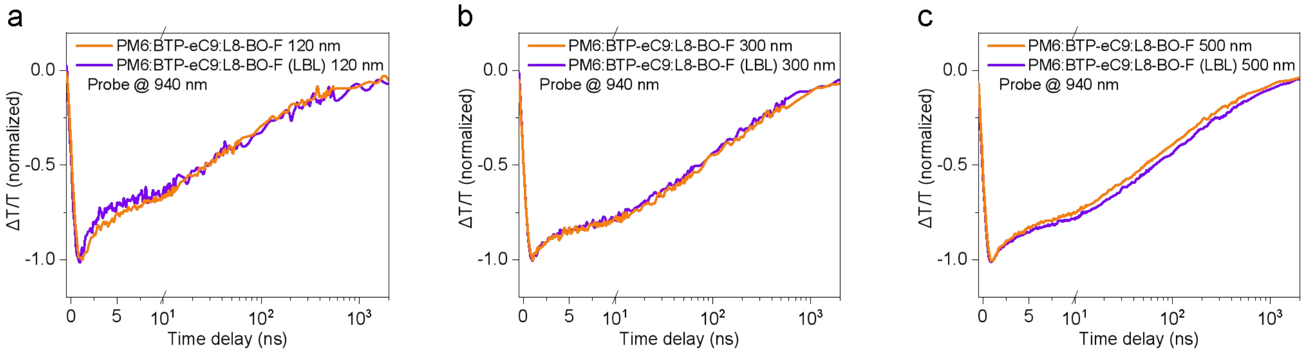

Supplementary Figure 22. Comparison of the free carrier (940 nm) dynamics of the samples with thicknesses of **a** 120 nm, **b** 300 nm, and **c** 500 nm. For the sample with thickness of 500 nm, the measurements were repeated over 20 times to improve the signal-to-noise ratio with limited transmitted probe light.

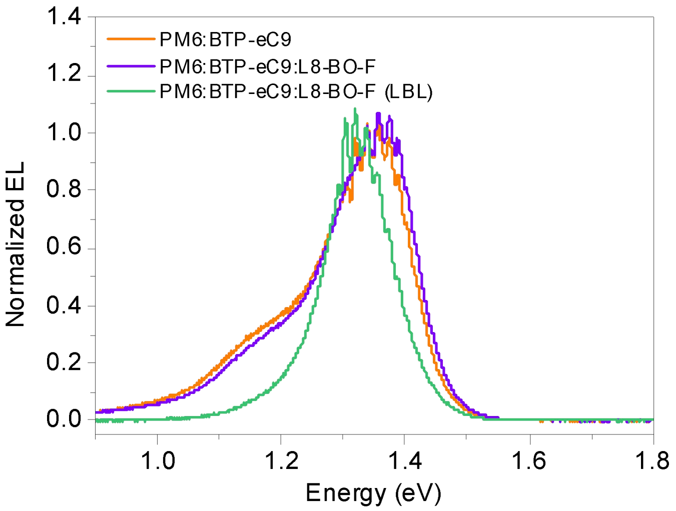

Supplementary Figure 23. Normalized EL curves of the PM6:BTP-eC9 and PM6:BTP-eC9:L8-BO-F blends.

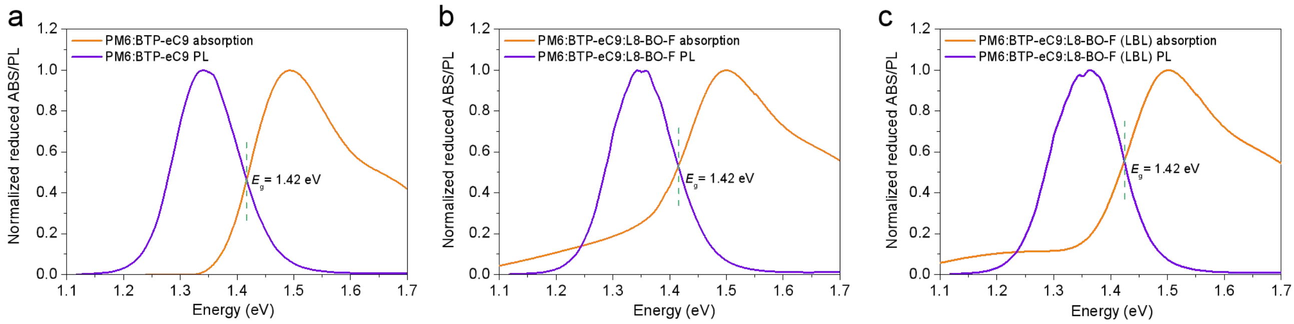

Supplementary Figure 24. Normalized reduced absorption and PL spectra of **a** PM6:BTP-eC9, **b** PM6:BTP-eC9:L8-BO-F, and **c** LBL-processed PM6:BTP-eC9:L8-BO-F films.

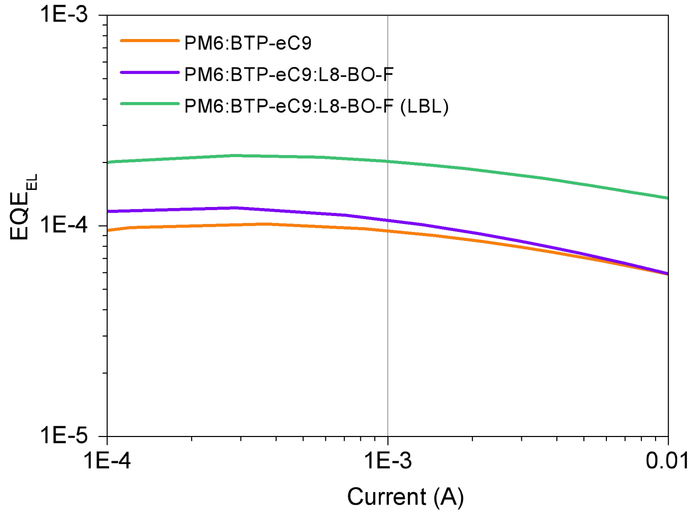

Supplementary Figure 25. EQE_{EL} spectra of the PM6:BTP-eC9 and PM6:BTP-eC9:L8-BO-F blends.

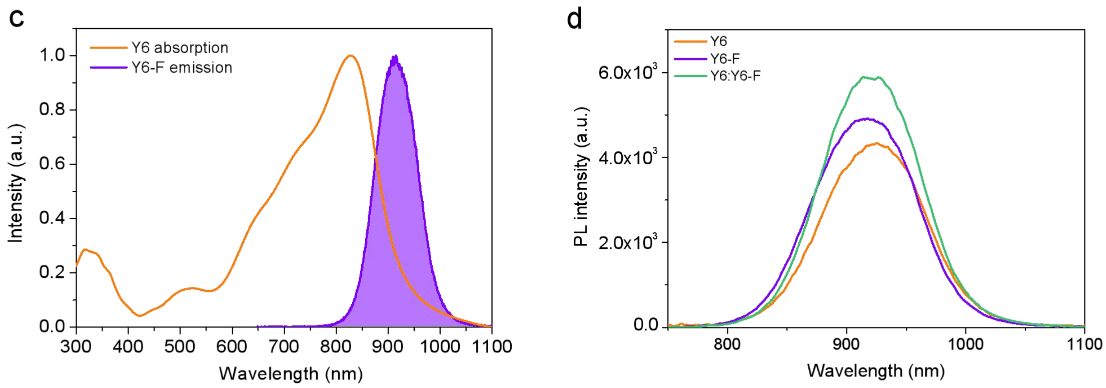

Supplementary Figure 26. a UV absorption spectrum of Y6 and PL spectrum of Y6-F. **b** PL spectra of Y6, Y6-F and Y6:Y6-F films.

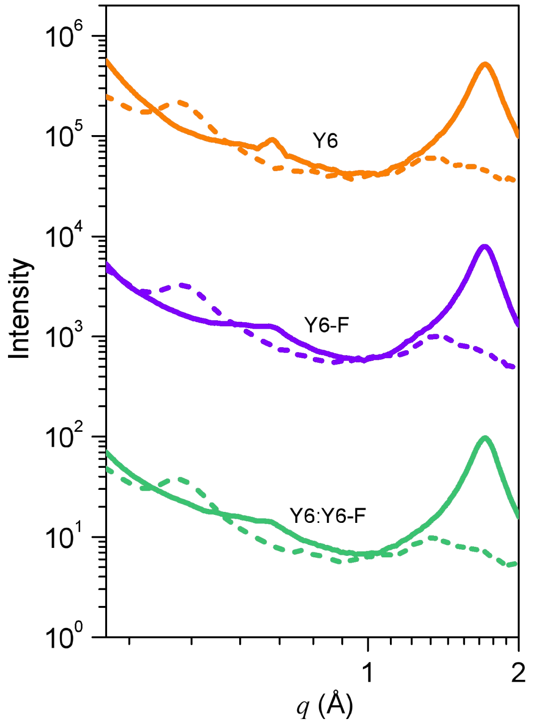

Supplementary Figure 27. In-plane (dashed lines) and out-of-plane (solid lines) line-cuts extracted from the 2D GIWAXS profiles of neat Y6, Y6-F and Y6:Y6-F films.

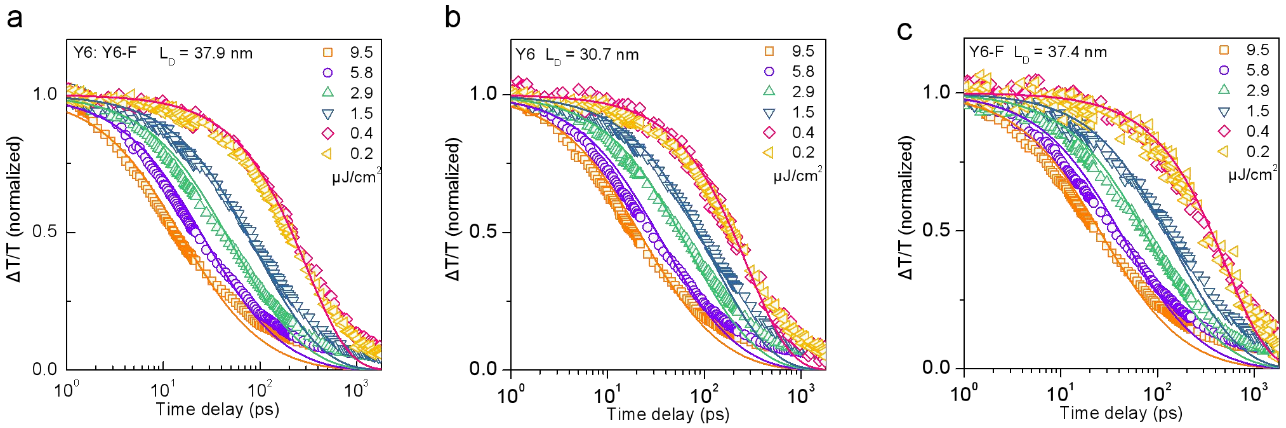

Supplementary Figure 28. The dynamics of the singlet excitons measured at different densities in films of **a** Y6:Y6-F (850 nm), **b** Y6 (850 nm), **c** Y6-F (830 nm).The fluence-dependent singlet exciton decays are fitted to the exciton annihilation model (Eq. (1) in the main text).

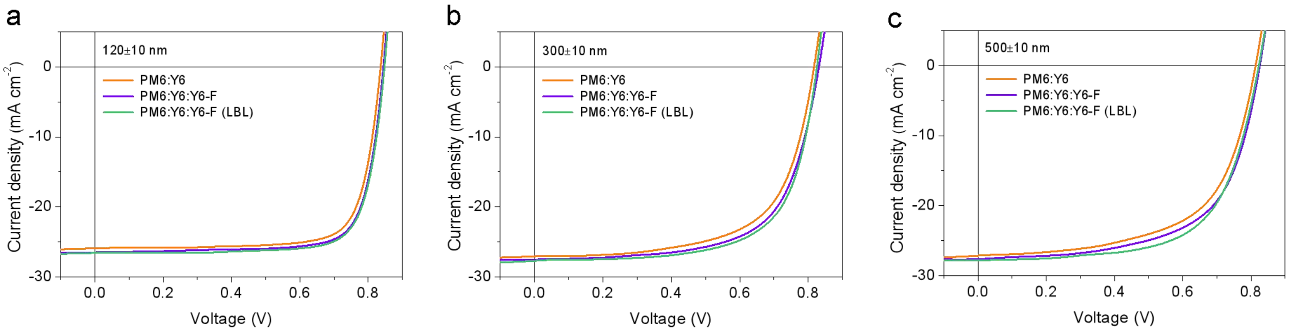

Supplementary Figure 29. *J-V* characteristics of PM6:Y6 and PM6:Y6:Y6-F devices with active layer thicknesses of **a** 120 nm, **b** 300 nm, and **c** 500 nm under simulated AM 1.5 G illumination at 100 mWcm^{-2}.

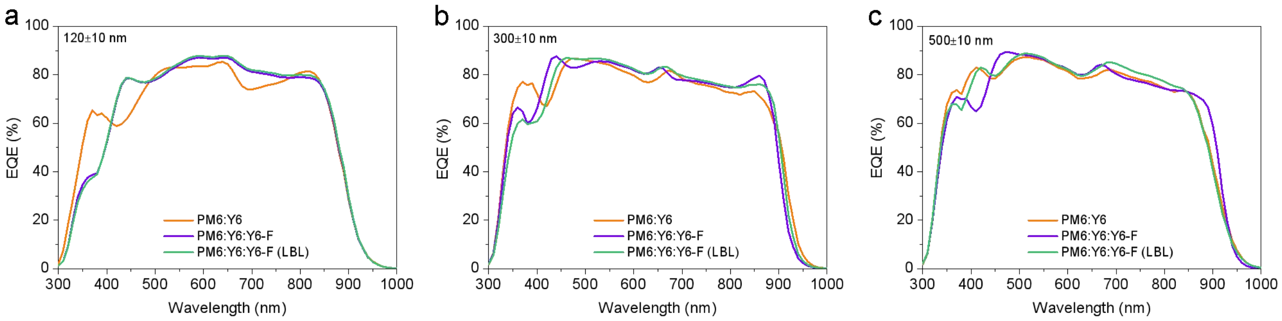

Supplementary Figure 30. EQE curves of PM6:Y6 and PM6:Y6:Y6-F devices with active layer thicknesses of **a** 120 nm, **b** 300 nm, and **c** 500 nm.

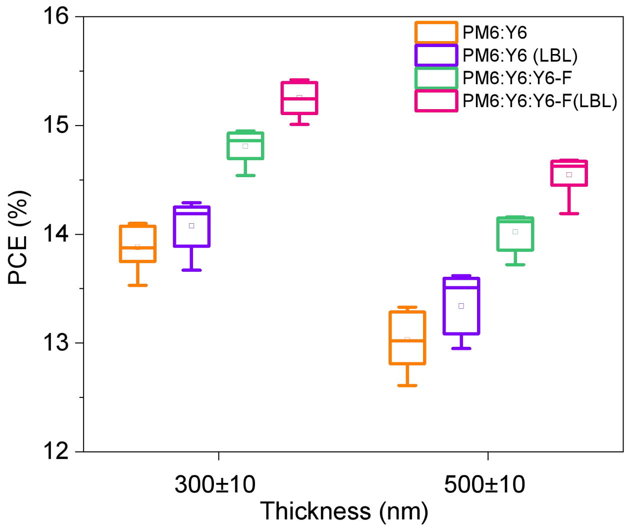

Supplementary Figure 31. Comparison of PCEs of the conventional and LBL-processed PM6:Y6 and PM6:Y6:Y6-F devices with different thicknesses. The box, horizontal line, and square symbol denote 25/75 percentile, the median value, and mean value respectively. The sample size for each column is 20 devices.

Supplementary Table 1. Detailed device parameters of reported representative thick-film OSCs.

Active layer	Thickness (nm)	V_{oc} (V)	J_{sc} (mA cm^{-2})	FF (%)	PCE (%)	Ref.
P4TNTz-2F:$PC_{71}BM$	350	0.82	19.45	6.5	10.62	1
PFBT4T-C5Si-25%:$PC_{71}BM$	420	0.76	19.08	74.12	11.09	2
PM6:IT4F	285	0.83	22.6	64.8	12.2	3
PM7:MF2	500	0.953	19.20	54.9	10.04	4
PTQ10:IDTPC	400	0.913	17.90	61.3	10.0	5
Si25:IEICO-4F	320	0.70	26.87	70.15	13.2	6
PT2:TTPTTT-4F	400	0.87	20.5	56.5	10.1	7
	500	0.87	20.8	53.5	9.7	7
PBDB-T-2Cl:BTP-4F	300	0.821	27.6	54.1	12.2	8
PBDB-T:PJ1	305	0.87	21.1	65	12.1	9
PNTT:BTR: $PC_{71}BM$	280	0.75	20.88	70.67	11.44	10
PT2:TTPTTT-4F:IDIC	300	0.87	20.6	66.7	12.2	7
	400	0.86	21.5	65.3	12.4	7
	500	0.86	22.0	61.4	11.6	7
PBDB-T-2Cl:BTP-4F: $PC_{61}BM$	300	0.802	26.8	66.7	14.3	8
PBDB-T-2Cl:BP-4F:MF1	300	0.882	23.06	71.62	14.57	11
PM6:Y6:BTP-M	300	0.855	26.87	62.06	14.23	12
BTR:NITI: $PC_{71}BM$	300	0.94	19.50	73.83	13.63	13
PM6:F-2Cl	350	0.866	19.73	58	10.00	14
	500	0.852	19.78	53	9.13	14
PffBT4T-2OD: $PC_{71}BM$	300	0.77	18.8	75	10.80	15
Si25:Y14	320	0.782	24.92	74.69	14.55	16
	390	0.782	24.53	73.68	14.79	16
	430	0.782	25.57	73.38	15.39	16
	480	0.782	26.29	71.87	15.26	16
PNTT: $PC_{71}BM$	280	0.77	20.20	71.8	11.3	17
PBDB-T-2Cl:Y6: $PC_{61}BM$	300	0.802	26.8	66.7	14.3	8
PFBT4T-C5Si-25%:O-IDTBR	400	1.03	21.1	53.07	11.54	18
P2:IT4F:BTP-4Cl	300	0.87	21.98	70	12.98	19
PBDB-TF:IDIC-C5Ph	470	0.921	20.15	70.12	13.01	20
D18:Y6	300	0.844	26.13	69.1	15.24	21
	350	0.845	26.26	67.8	15.04	21
D18:Y6: $PC_{61}BM$	300	0.86	26.15	72.6	16.32	21
	350	0.861	26.82	70.1	16.19	21

Supplementary Table 2. Summary of photovoltaic parameters of PM6:BTP-eC9 devices with different active layer thicknesses under different processing conditions.

Active layer	Thickness (nm)	V_{oc} (V)	J_{sc} (mA cm^{-2})	FF (%)	PCE[a] (%)
PM6:BTP-eC9	120±10	0.840 (0.839 ±0.002)	26.61 (26.30±0.30)	78.2 (77.5±0.6)	17.47 (17.28±0.17)
	300±10	0.820 (0.819±0.002)	27.64 (27.35±0.28)	68.9 (68.2±0.6)	15.62 (15.40±0.18)
	500±10	0.818 (0.817±0.002)	26.13 (25.81±0.31)	61.5 (61.1±0.5)	13.14 (12.95±0.17)
PM6:BTP-eC9 (LBL)	120±10	0.839 (0.838±0.001)	26.63 (26.21±0.45)	78.5 (77.8±0.6)	17.54 (17.23±0.30)
	300±10	0.819 (0.817±0.002)	27.78 (27.30±0.49)	70.6 (70.1±0.4)	16.02 (15.95±0.16)
	500±10	0.818 (0.817±0.002)	26.61 (26.31±0.32)	63.3 (62.5±0.7)	13.78 (13.56±0.21)

[a]Average values with standard deviation were obtained from 40 devices.

Supplementary Table 3. Detailed parameters of single exciton decay dynamic for neat and mixed acceptor films.

**Materials**	**κ (10^{-3} ps^{-1})**	**α (nm^3ps^{-1})**	**τ (ps)**	**D (nm^2ps^{-1})**	**L_D (nm)**
BTP-eC9	5.36 ± 0.18	182.2 ± 2.2	187	7.17	36.6
L8-BO-F	3.37 ± 0.12	151.7 ± 2.1	303	6.04	44.4
BTP-eC9:L8-BO-F	3.30 ± 0.07	182.9 ± 2	297	7.28	47.0

Supplementary Table 4. Crystal coherence lengths of the (010) peak and the d-spacing for neat BTP-eC9, L8-BO-F and BTP-eC9:L8-BO-F blends.

Active layer	q_z (Å^{-1})	d-spacing (Å)	FWHM (Å^{-1})	CCL (Å)
BTP-eC9	1.711	3.672	0.306	18.54
L8-BO-F	1.693	3.711	0.351	16.11
BTP-eC9:L8-BO-F	1.705	3.685	0.295	19.17

Supplementary Table 5. Crystal coherence lengths of the (010) peak and the *d*-spacing for the PM6:BTP-eC9 and PM6:BTP-eC9:L8-BO-F blend films.

Active layer	q_z (Å^{-1})	d-spacing (Å)	FWHM (Å^{-1})	CCL (Å)
PM6:BTP-eC9	1.707	3.681	0.243	23.012
PM6:BTP-eC9:L8-BO-F	1.704	3.687	0.240	23.300
PM6:BTP-eC9:L8-BO-F(LBL)	1.703	3.690	0.235	23.796

Supplementary Table 6. Crystal coherence lengths of the (100) peak and the *d*-spacing for the PM6:BTP-eC9 and PM6:BTP-eC9:L8-BO-F blends.

Active layer	q_{xy} ($Å^{-1}$)	d-spacing (Å)	FWHM ($Å^{-1}$)	CCL (Å)
PM6:BTP-eC9	0.296	21.227	0.060	93.201
PM6:BTP-eC9:L8-BO-F	0.296	21.227	0.055	101.673
PM6:BTP-eC9:L8-BO-F (LBL)	0.295	21.299	0.052	107.539

Supplementary Table 7. Detailed energy loss of OSCs based on PM6:BTP-eC9 and PM6:BTP-eC9:L8-BO-F blends.

**Active layer**	V_{oc} [V]	E_g [a] [eV]	V_{loss} [V]	ΔV_{rad} [b] [V]	$\Delta V_{non\text{-}rad}$ [c] [V]	**EQE$_{EL}$**
PM6:BTP-eC9	0.82	1.42	0.60	0.37	0.23	9.5×10^{-5}
PM6:BTP-eC9:L8-BO-F	0.84	1.42	0.58	0.35	0.23	1.1×10^{-4}
PM6:BTP-eC9:L8-BO-F (LBL)	0.84	1.42	0.58	0.37	0.21	2.0×10^{-4}

[a] E_g is the optical bandgap of the film determined from the normalized reduced absorption and PL spectra of films.
[b] ΔV_{rad} is the voltage loss associated with radiative recombination.
[c] $\Delta V_{non\text{-}rad}$ is the voltage loss associated with non-radiative recombination.

Supplementary Table 8. Crystal coherence lengths of the (010) peak and the d-spacing for neat Y6, Y6-F and Y6:Y6-F blends.

Active layer	q_z (Å^{-1})	d-spacing (Å)	FWHM (Å^{-1})	CCL (Å)
Y6	1.709	3.676	0.286	19.77
Y6-F	1.703	3.689	0.272	20.79
Y6:Y6-F	1.706	3.682	0.263	21.50

Supplementary Table 9. Detailed parameters of single exciton decay dynamic for neat and mixed acceptor films.

Materials	κ (10^{-3} ps^{-1})	α ($nm^3 ps^{-1}$)	τ (ps)	D ($nm^2 ps^{-1}$)	L_D (nm)
Y6	3.50 ± 0.03	82.82 ± 0.9	286	3.30	30.7
Y6-F	1.94 ± 0.02	68.7 ± 0.7	515	2.73	37.4
Y6:Y6-F	3.26 ± 0.03	117.4 ± 1.1	307	4.67	37.9

Supplementary Table 10. Summary of photovoltaic parameters of PM6:Y6, and PM6:Y6:Y6-F devices with different active layer thicknesses.

Active layer	Thickness (nm)	V_{oc} (V)	J_{sc} (mA cm^{-2})	FF (%)	PCE[a] (%)
PM6:Y6	120±10	0.841 (0.840±0.002)	25.85 (25.35±0.51)	77.3 (76.8±0.6)	16.80 (16.55±0.25)
	300±10	0.818 (0.816±0.002)	27.02 (26.71±0.29)	63.8 (63.3±0.6)	14.10 (13.90±0.16)
	500±10	0.815 (0.813±0.002)	27.13 (26.75±0.39)	60.3 (59.5±0.7)	13.33 (13.10±0.24)
PM6:Y6 (LBL)	120±10	0.840 (0.839±0.002)	25.76(25.33±0.45)	78.0(79.5±0.4)	16.88(16.70±0.20)
	300±10	0.815 (0.813±0.002)	27.16(26.85±0.33)	64.6(67.2±0.5)	14.29(14.03±0.26)
	500±10	0.813 (0.812±0.002)	27.42(27.00±0.46)	61.2(62.2±0.6)	13.64(13.30±0.33)
PM6:Y6:Y6-F	120±10	0.852 (0.851±0.001)	26.50 (26.12±0.37)	77.5 (77.1±0.5)	17.50 (17.36±0.15)
	300±10	0.832 (0.829±0.002)	27.49 (27.08±0.40)	65.4 (64.7±0.6)	14.96 (14.80±0.16)
	500±10	0.827 (0.824±0.002)	27.66 (27.34±0.31)	61.9 (61.3±0.6)	14.16 (14.00±0.17)
PM6:Y6:Y6-F (LBL)	120±10	0.851 (0.850±0.002)	26.56 (26.25±0.32)	77.8 (77.1±0.6)	17.58 (17.42±0.17)
	300±10	0.828 (0.827±0.002)	27.67 (27.38±0.29)	67.3 (66.8±0.6)	15.42 (15.27±0.16)
	500±10	0.823 (0.822±0.002)	27.78 (27.37±0.42)	64.3 (63.8±0.5)	14.70 (14.53±0.18)

[a]Average values with standard deviation were obtained from 40 devices.

Supplementary References

1. Lee, J. et al. Highly crystalline low-bandgap polymer nanowires towards high-performance thick-film organic solar cells exceeding 10% power conversion efficiency. *Energy Environ. Sci.* **10**, 247–257 (2017).
2. Liu, X. et al. Low band gap conjugated polymers combining siloxane-terminated side chains and alkyl side chains: side-chain engineering achieving a large active layer processing window for PCE > 10% in polymer solar cells. *J. Mater. Chem. A* **5**, 17619–17631(2017).
3. Fan, Q. et al. Synergistic effect of fluorination on both donor and acceptor materials for high performance non-fullerene polymer solar cells with 13.5% efficiency. *Sci. China Chem.* **61**, 531–537 (2018).
4. Gao, W. et al. Thick-Film Organic Solar Cells Achieving over 11% Efficiency and Nearly 70% Fill Factor at Thickness over 400 nm. *Adv. Funct. Mater.* **30**, 1908336 (2020).
5. Luo, Z. et al. Side-chain impact on molecular orientation of organic semiconductor acceptors: high performance nonfullerene polymer solar cells with thick active layer over 400 nm. *Adv. Energy Mater.* **8**, 1800856(2018).
6. Wang, Z. et al. Significantly enhanced electron transport of a nonfullerene acceptor in a blend film with a high hole mobility polymer of high molecular weight: thick-film nonfullerene polymer solar cells showing a high fill factor. *J. Mater. Chem. A* **8**, 7765–7774 (2020).
7. Weng, K. et al. High-efficiency organic solar cells with wide toleration of active layer thickness. *Sol. RRL* **4**, 2000476 (2020).
8. Ma, L. et al. A ternary organic solar cell with 300 nm thick active layer shows over 14% efficiency. *Sci. China Chem.* **63**, 21–27 (2019).
9. Jia, T. et al. 14.4% efficiency all-polymer solar cell with broad absorption and low energy loss enabled by a novel polymer acceptor. *Nano Energy* **72**, 104718 (2020).
10. Xiao, M. et al. Low temperature processed high-performance thick film ternary polymer solar cell with enhanced stability. *Nano Energy* **48**, 53–62 (2018)
11. Gao, J. et al. Over 14.5% efficiency and 71.6% fill factor of ternary organic solar cells with 300 nm thick active layers. *Energy Environ. Sci.* **13**, 958–967 (2020).
12. Zhan, L. et al. Over 17% efficiency ternary organic solar cells enabled by two non-fullerene acceptors working in an alloy-like model. *Energy Environ. Sci.* **13**, 635–645(2020).
13. Zhou, Z. et al. High-efficiency small-molecule ternary solar cells with a hierarchical morphology enabled by synergizing fullerene and non-fullerene acceptors. *Nat. Energy* **3**, 952–959 (2018).
14. Zhang, Y. et al. High performance thick-film nonfullerene organic solar cells with efficiency over 10% and active layer thickness of 600 nm. *Adv. Energy Mater.* **9**, 1902688 (2019).
15. Liu, Y. et al. Aggregation and morphology control enables multiple cases of high-efficiency polymer solar cells. *Nat. Commun.* **5**, 5293 (2014).
16. Pan, F. et al. Binary nonfullerene polymer solar cells with 430 nm thick active layer showing 15.39% efficiency and 73.38% fill factor. *J. Mater. Chem. A* **9**, 7129-7136 (2021).
17. Jin, Y. et al. Thick Film Polymer Solar Cells Based on Naphtho[1,2-c:5,6-c]bis[1,2,5]thiadiazole Conjugated Polymers with Efficiency over 11%. *Adv. Energy Mater.* **7**, 1700944 (2017).
18. Wang, Z. et al. Organic solar cells based on high hole mobility conjugated polymer and nonfullerene acceptor with comparable bandgaps and suitable energy level offsets showing significant suppression of J_{sc}-V_{oc} trade-off. *Solar RRL* **3**,100079 (2019).
19. Gokulnath, T. et al. Nonhalogenated solvent-processed thick-film ternary nonfullerene organic solar cells with power conversion efficiency >13% enabled by a new wide-bandgap polymer. *Sol. RRL* 2000787 (2021).
20. Li, Y. et al. Subtle side chain triggers unexpected two-channel charge transport property enabling 80% fill

factors and efficient thick-film organic photovoltaics. *The innovation* **2**, 100090 (2021).

21. Qin, J. et al. Over 16% efficiency from thick-film organic solar cells. *Sci. Bull.* **65**, 1979–1982 (2020).
